# Correlating plasma protein profiles with symptomatology and treatment response in acute phase and early remission of major depressive disorder

**DOI:** 10.3389/fpsyt.2024.1425552

**Published:** 2024-09-17

**Authors:** Pavel Křenek, Eliška Bartečková, Markéta Makarová, Tomáš Pompa, Jana Fialová Kučerová, Jan Kučera, Alena Damborská, Jana Hořínková, Julie Bienertová-Vašků

**Affiliations:** ^1^ Department of Psychiatry, Faculty of Medicine, Masaryk University and University Hospital Brno, Brno, Czechia; ^2^ Department of Physical Activities and Health Sciences, Faculty of Sport Science, Masaryk University, Brno, Czechia; ^3^ Department of Pathological Physiology, Faculty of Medicine, Masaryk University, Brno, Czechia

**Keywords:** major depressive disorder, plasma proteomics, LC-MS/MS, immune response, symptom presentation, treatment response, biomarker in depression

## Abstract

**Objectives:**

This study aimed to explore the relationship between plasma proteome and the clinical features of Major Depressive Disorder (MDD) during treatment of acute episode.

**Methods:**

In this longitudinal observational study, 26 patients hospitalized for moderate to severe MDD were analyzed. The study utilized Liquid Chromatography with Tandem Mass Spectrometry (LC-MS/MS) alongside clinical metrics, including symptomatology derived from the Montgomery-Åsberg Depression Rating Scale (MADRS). Plasma protein analysis was conducted at the onset of acute depression and 6 weeks into treatment. Analytical methods comprised of Linear Models for Microarray Data (LIMMA), Weighted Correlation Network Analysis (WGCNA), Generalized Linear Models, Random Forests, and The Database for Annotation, Visualization and Integrated Discovery (DAVID).

**Results:**

Five distinct plasma protein modules were identified, correlating with specific biological processes, and uniquely associated with symptom presentation, the disorder’s trajectory, and treatment response. A module rich in proteins related to adaptive immunity was correlated with the manifestation of somatic syndrome, treatment response, and inversely associated with achieving remission. A module associated with cell adhesion was linked to affective symptoms and avolition, and played a role in the initial episodes and treatment response. Another module, characterized by proteins involved in blood coagulation and lipid transport, exhibited negative correlations with a variety of MDD symptoms and was predominantly associated with the manifestation of psychotic symptoms.

**Conclusion:**

This research points to a complex interplay between the plasma proteome and MDD’s clinical presentation, suggesting that somatic, affective, and psychotic symptoms may represent distinct endophenotypic manifestations of MDD. These insights hold potential for advancing targeted therapeutic strategies and diagnostic tools.

**Limitations:**

The study’s limited sample size and its naturalistic design, encompassing diverse treatment modalities, present methodological constraints. Furthermore, the analysis focused on peripheral blood proteins, with potential implications for interpretability.

## 1 Introduction

Major Depressive Disorder (MDD) stands as a serious public health challenge of our era. With an annual prevalence of major depressive episodes affecting approximately 6% of the global population and a lifetime prevalence of around 11% in the developing world and 15% in high-income countries, depressive disorders rank among the top three leading causes of Years Lived with Disability (YLDs), exacting an enormous toll on individuals and society (1, 2).

Current research in psychiatry continues to investigate the pathophysiology of depression, focusing on the wide array of biochemical processes that underlie the presentation of MDD and its progression to a recurrent, neuroprogressive course. These processes include the activation of immune-inflammatory pathways, as demonstrated by a broad range of changes in both innate and adaptive immunity, and oxidative stress mechanisms, as evidenced by increased lipid peroxidation, oxidative damage to DNA and mitochondria, membrane damage, and lowered antioxidant levels (3–6). Despite these insights, the causes of MDD remain not fully understood (7).

Proteomics has become a key area in the search for accurate biomarkers, offering a comprehensive way to study proteins in biological systems (8). Specifically, analyzing the human blood proteome has attracted significant attention because it allows for non-invasive testing and holds potential for discovering indicators that reflect the intricate neurobiology of MDD (9).

In the field of blood-based proteomic research on MDD, existing studies have provided insights into abnormal protein expression profiles indicative of the disorder. These profiles suggest alterations in several biological pathways including inflammation, coagulation, energy metabolism, oxidative stress, neuroplasticity, and neurotransmitter systems. However, the field faces challenges with consensus and reproducibility, highlighting the need for efforts to reconcile these findings (9–11).

Consistent with the framework proposed by the National Institute of Mental Health’s Research Domain Criteria matrix, there’s an increasing emphasis on biologically stratifying patients into more homogenous and clinically distinct subgroups (12). This strategy aims to address the diverse manifestations of MDD and could lay the groundwork for more personalized and effective treatments.

For these reasons, we sought to investigate the correlations between protein expression and the manifestation of specific symptoms and subtypes of MDD. Given that the use of the established classification into melancholic and atypical depression is constrained by the prevalence of these subtypes—with less than 50% of patients showing definitive signs of either subtype, while the majority exhibit mixed features of both — we have chosen to draw inspiration from the constructs of depression core features postulated by Dooley et al. (2018) and the clinically defined depression subtypes established and validated by Sharpley and Bitsika (13–16).

Dooley et al. (2018) proposed four core features of depressive symptoms: exaggerated reactivity to negative information, altered reward processing, cognitive control deficits, and somatic syndrome. Sharpley and Bitsika (2013) outlined four clinical content subtypes: depressed mood, anhedonia, cognitive depression, and somatic depression. These clusters of symptoms overlap and partially correspond to four of six domains of the Research Domain Criteria (12). The extensive details of these clusters were elaborated in the works of Dooley et al. (2018) and Sharpley and Bitsika (2013), and discussed in our previous review (14, 15, 17).

This study aimed to evaluate the diagnostic and prognostic potential of plasma protein expression measured via Liquid Chromatography Tandem Mass Spectrometry (LC-MS/MS) in relation to depressive symptoms during the acute phase and early remission of MDD in hospitalized patients. Particular objectives were:

- To identify specific proteins and clusters of highly co-expressed proteins in the plasma of MDD patients, and characterize the biological processes linked to these proteins.
- To assess whether the plasma protein expression is associated with the overall severity of MDD, and severity of specific MDD symptoms and symptom clusters derived from the Montgomery-Åsberg Depression Rating Scale (MADRS).
- To evaluate whether plasma protein expression is associated with the reduction in MDD severity, treatment response, and achieving remission.

## 2 Materials and methods

In total, 26 subjects of Central European ancestry, hospitalized for moderate or severe depressive episode, were enrolled into the study. Measurements were carried out at two time points - week 0, characterized by acute depression, and week 6, six weeks after enrollment in the study.

The Ethics Committee of the University Hospital Brno thoroughly reviewed and approved the study protocol, as well as the methodologies employed for sample collection and analysis. All participants provided written informed consent after receiving a comprehensive explanation of the study. All procedures were conducted in accordance with the principles outlined in the Helsinki Declaration.

### 2.1 Subjects

Patients were recruited from acute wards at the Department of Psychiatry, University Hospital Brno, Czech Republic.

The study included patients aged 18 to 65 years, experiencing moderate to severe depressive episodes with or without psychotic symptoms, falling within the spectrum of MDD. Each patient underwent a two-step diagnostic process: First, a board-certified psychiatrist established a diagnosis according to International Classification of Diseases, 10th Revision (diagnosis of F32.1-F32.3 or F33.1-F33.3) (18). Next, the diagnosis was confirmed by a second psychiatrist using the Czech version of The Mini-International Neuropsychiatric Interview (MINI) (19). Severity of depressive symptoms was further assessed by the MADRS with a minimum score of 18 points (20).

Exclusion criteria included psychiatric comorbidities (with the exception of simple psychoactive substance abuse without dependency syndrome and specific anxiety disorders, including generalized anxiety disorder, panic disorder, and phobic disorders, as well as less severe personality disorders). Another exclusion criteria were an IQ below 70, significant findings on brain magnetic resonance imaging, pregnancy, the postpartum period, decompensated or only partially compensated somatic comorbidities (such as cancer, cardiovascular, metabolic, endocrine, infectious, autoimmune, degenerative, traumatic, and functional disorders), noncompliance, diminished capacity for voluntary consent, and involuntary hospitalization.

Patients were recruited into the study within the first two weeks of hospitalization and treatment, or when transitioning to a different treatment modality due to ineffectiveness of previous treatment. Patients were treated as usual with standard biological modalities (antidepressants, antipsychotics, mood stabilizers, anxiolytics, brain stimulation therapies), and participation in the study did not influence treatment choice or the length of hospitalization.

### 2.2 Clinical variables

Clinical outcomes were assessed through psychiatric examinations, using the structured interview MINI (conducted at week 0), and the MADRS scale (measured at week 0 and week 6). The diagnoses of F32.1-F32.3 or F33.1-F33.3 were established by standard clinical assessment. The severity of depression was measured as the total MADRS score, treatment response as a reduction of MADRS by 50% or more, and achieving remission was defined as MADRS scores less than 10 points (21). The first episode was represented by the diagnosis F32.1-F32.3. Psychotic symptoms were assessed at week 0 through clinical examination and the MINI.

In accordance with the work of Sharpley and Bitsika (15) and Dooley et al. (14), depressive symptoms assessed by MADRS were mapped into four clusters representing different putative neurobiological underpinnings of depression:

- Exaggerated reactivity to negative information (MADRS cluster A): Reported sadness (MADRS 1), inner tension (MADRS 3), pessimistic thoughts (MADRS 9), and suicidal thoughts (MADRS 10)
- Altered reward processing (MADRS cluster B): Inability to feel (MADRS 8)
- Deficits in cognitive control (MADRS cluster C): Concentration difficulty (MADRS 6)
- Somatic syndrome (MADRS cluster D): Reduced sleep (MADRS 4), reduced appetite (MADRS 5), and lassitude (MADRS 7)

Apparent sadness (MADRS 2) was excluded from this categorization because, being defined as “despondency, gloom, and despair (more than just ordinary transient low spirits), reflected in speech, facial expression, and posture; rate by depth and inability to brighten up,” it could represent both exaggerated reactivity to negative information and altered reward processing.

In addition to the aforementioned theoretical clusters, three empirical MADRS clusters derived from Principal Component Analysis, as elucidated by Suzuki et al. (2005), were incorporated into our research (22). These clusters, which collectively accounted for 61% of the total variance, were delineated as follows:

- Dysphoria (MADRS dysphoria): Reported sadness (MADRS 1), pessimistic thoughts (MADRS 9), and suicidal thoughts (MADRS 10)
- Retardation (MADRS retardation): Apparent sadness (MADRS 2), concentration difficulty (MADRS 6), lassitude (MADRS 7), and inability to feel (MADRS 8)
- Vegetative symptoms (MADRS vegetative): Inner tension (MADRS 3), reduced sleep (MADRS 4), reduced appetite (MADRS 5)

### 2.3 Blood samples and proteomic experiments

Blood samples were taken after an overnight fast, immediately transferred to a laboratory of the Department of Pathological Physiology (Faculty of Medicine, Masaryk University, Brno, Czech Republic) and centrifuged 10 min at 2500 g at room temperature to separate plasma. All plasma samples were aliquoted and stored at -80°C within 2 hours from collection. Proteomic analysis was performed using label-free quantification by LC-MS/MS. LC-MS/MS system was composed of an Ultimate 3000 RSLCnano system (SRD-3400, NCS-3500RS CAP, WPS-3000 TPL RS; Thermo Fisher Scientific) integrated with an Orbitrap Fusion Lumos system (Thermo Fisher Scientific) equipped with a Digital PicoView 550 nanospray ion source. Tryptic digests, approximately 2 µg per injection, were first concentrated and desalted online on a trapping column (100 μm × 30 mm, packed with 3.5 μm X-Bridge BEH 130 C18 sorbent from Waters, Milford) using 0.1% formic acid in water. The peptides were then transferred from the trapping column to an Acclaim Pepmap100 C18 analytical column (3 µm particles, 75 μm × 500 mm; Thermo Fisher Scientific, Cat# 164570). A 120-minute gradient was employed for peptide separation, using mobile phase A (0.1% formic acid in water) and mobile phase B (0.1% formic acid in 80% (v/v) acetonitrile). The peptides were eluted using a linear gradient of 1% to 30% mobile phase B over 75 minutes, followed by an increase to 56% over 30 minutes, then a 5-minute increase to 80%, and finally a 10-minute wash with 80% mobile phase B, all at a flow rate of 300 nL/min.

Mass spectrometry data were collected through data-dependent acquisition. The target parameters for full scan MS spectra included a charge of 4 × 10^5 in the m/z range of 350 to 2000, with a maximum injection time of 54 ms and a resolution of 60,000 at m/z 200. MS/MS scans were conducted post-HCD fragmentation using 30% collision energy, at a resolution of 30,000 at m/z 200, with an ion target value of 5 × 10^4 and a maximum injection time of 50 ms. All peptide mixtures were analyzed separately in a randomized order, week 0 and week 6 samples from each patient were analyzed together.

### 2.4 Statistical and bioinformatics analyses

This investigation employed Linear Models for Microarray Data (LIMMA), Weighted Correlation Network Analysis (WGCNA), Generalized Linear Models (GLMs), and Random Forests (RFs) for statistical analysis to investigate associations between protein intensities and MADRS clusters (23–26). Covariates consisted of sex, age, and body mass index (BMI). Functional annotation was performed using The Database for Annotation, Visualization and Integrated Discovery (DAVID) tool (27).

Protein intensities were processed using the R software (v4.2.0) (28). Full pre-processing workflow with attached code is available upon request. In summary, we

1. excluded patients without proteomic records,
2. transformed normalized and imputed intensities using a binary logarithmic transformation,
3. imputed two missing BMI values by a mean value due to the symmetric distribution of the present values.

The imputed normalized protein intensities were first analyzed using the differential expression method via the limma R package (29). Both paired and unpaired models were considered. Subsequently, the results were adjusted for multiple hypothesis testing using the Benjamini-Hochberg procedure (30).

Following that, we performed a global correlation analysis on the normalized imputed binary logarithmic transformed protein intensity data using the weighted gene co-expression network analysis, WGCNA (24, 31). We constructed a signed correlation consensus co-expression network using Pearson’s correlation. Pearson’s correlation was chosen over bi-weighted mid-correlation due to a relatively small sample size. The power of the model was chosen such that it satisfied two key criteria. Firstly, it approximately satisfied the scale-free topology criterion by satisfying R^2> 0.9 for both groups, where R^2 denotes scale-free topology model fit. Secondly, it was the smallest power that satisfied such a criterion. Singular signed correlation co-expression networks for week 0 and week 6 were constructed similarly. The adjacency matrices of these networks were used as a basis for hierarchical clustering of the proteins, resulting in clusters of highly intercorrelated proteins. A dimension reduction technique was deployed to represent each cluster of proteins by its first eigenvector, corresponding to the largest eigenvalue.

The relationships between protein clusters, represented by corresponding eigenvectors, and continuous clinical traits were determined by Peason’s correlation coefficient. Furthermore, the resulting correlation was tested for significance via Fishers Z-test.

The relationships between cluster eigenvectors and categorical clinical variables were investigated by GLMs and RFs. GLMs were fitted using lme4 R package (32). A given categorical clinical variable was modeled using consensus eigenvectors as explanatory variables. RFs models were fitted via Python scikit-learn package (33). The Python release used is 3.11.0. Similarly, the investigated categorical clinical variable acted as a target and the eigenvectors were used as explanatory variables. The hyperparameters (total number of trees, maximal depth, minimal number of samples in a split, minimal number of samples in a leaf) were tuned using a grid search cross-validation procedure. The final model performance was measured by accuracy and the effect of explanatory variables was judged by feature importance, permutation importance and SHAP values (34, 35).

The DAVID tool, facilitating functional annotation of biological processes, was used on 27.12.2023 (27). Overrepresentation and fold enrichment analyses for all protein groups (PGs) were conducted against the human genome background, and for each WGCNA module against background of all PGs in this dataset. Assessment of results employed EASE scoring, and Benjamini-Hochberg corrections (30, 36).

## 3 Results

### 3.1 Sample characteristics

The study encompassed a sample of 26 individuals with MDD, consisting of 12 women (46.2%) and 14 men (53.8%), with a mean age of 50.2 years (SD=12.5). Of these, 8 participants (30.8%) experienced their first episode, and 18 participants (69.2%) experienced a recurrent depressive episode. After six weeks of treatment, 8 (30.8%) were classified as non-responders, and 18 (69.2%) were responders. Of responders, 3 (11.5% of the total sample) were partial responders, and 15 (57.7% of the total sample) achieved remission. Additional sample characteristics are detailed in **Table 1**, with an overview of psychiatric treatment provided in **Table 2**. Initially, at week 0, 9 patients (34.6%) were using non-psychiatric medication, which increased to 11 patients (42.3%) by week 6. The overview of non-psychiatric medication use is summarized in **Table 3**.

**Table 1:** Sample characteristics.

	Week 0		Week 6	
Female (n, %)	12	46.2	12	46.2
Age (mean, s.d.)	50.2	12.5	50.2	12.5
Body mass index (kg/m3)	26.3	4.4	26.5	4.6
MADRS score (mean, s.d.)	30.7	9.8	11.3	10.1
Psychotic symptoms (n, %)	11	42.3	0	0

**Table 2:** Number of patients treated by each therapy.

Type	Compound	Week 0	Week 6
Antidepressants	Agomelatine	2	4
Antidepressants	Citalopram	4	3
Antidepressants	Clomipramine	0	1
Antidepressants	Escitalopram	0	1
Antidepressants	Mirtazapine	5	8
Antidepressants	Sertraline	2	3
Antidepressants	Trazodone	1	1
Antidepressants	Venlafaxine	3	3
Antidepressants	Vortioxetine	7	7
Antipsychotics	Amisulpride	2	2
Antipsychotics	Aripiprazole	4	6
Antipsychotics	Clozapine	0	1
Antipsychotics	Olanzapine	8	7
Antipsychotics	Quetiapine	6	4
Antipsychotics	Tiapride	2	0
Antiparkinson drugs	Biperiden	0	1
Anxiolytics	Buspirone	0	1
Anxiolytics	Clonazepam	16	2
Anxiolytics	Oxazepam	4	5
Anxiolytics	Pregabalin	1	2
Anxiolytics	Promethazine	0	3
Hypnotics	Zolpidem	0	1
Mood stabilizers	Carbamazepine	1	0
Mood stabilizers	Lithium	0	1
Mood stabilizers	Valproate	0	1
Brain stimulation therapy	ECT	5	0
Brain stimulation therapy	rTMS	2	0
Brain stimulation therapy	Phototherapy	2	0

ECT, electroconvulsive therapy; rTMS, repetitive transcranial magnetic stimulation.

**Table 3:** Number of patients treated by non-psychiatric medication.

Type	Compound	Week 0	Week 6
Antihypertensives	Perindopril	4	4
Antihypertensives	Telmisartan	3	1
Antihypertensives	Amlodipine	2	2
Antihypertensives	Rilmenidine	1	0
Antihypertensives	Indapamide	1	0
Antihypertensives	Hydrochlorothiazide	1	1
Antihypertensives	Metoprolol	0	2
Hypolipidemics	Rosuvastatin	2	2
Thyroid Hormones	Levothyroxine	2	2
PPIs	Omeprazole	1	3
PPIs	Esomeprazole	1	1
Anticoagulants	Warfarine	1	1
Antiplatelets	Acetylsalicylic acid	1	1
BPH Therapies	Tamsulosin	1	1
Mineral Supplements	Potassium chloride	1	1

PPIs, Proton Pump Inhibitors; BPH, Benign Prostatic Hyperplasia.

### 3.2 Proteomic analysis

A comparative proteomic analysis, conducted using label-free quantification via LC-MS/MS, led to the identification of 812 PGs, corresponding to 947 plasma proteins. Functional annotations of biological processes associated with these PGs are described in **Table 4**.

**Table 4:** Biological processes of 812 protein groups detected from peripheral blood of patients with major depression, with overrepresentation test and fold enrichment using human genome as a background.

Biological Process	Protein Groups Count	Protein Groups Count (%)	EASE (P-Value)	Fold Enrichment	Benjamini (P-Value)
Immunity	156	19.6	<0.001	3.7	**<0.001**
Cell adhesion	81	10.2	<0.001	3.7	**<0.001**
Adaptive immunity	81	10.2	<0.001	3.6	**<0.001**
Innate immunity	70	8.8	<0.001	3.7	**<0.001**
Host-virus interaction	63	7.9	<0.001	2.1	**<0.001**
Blood coagulation	33	4.1	<0.001	14.7	**<0.001**
Hemostasis	33	4.1	<0.001	14.7	**<0.001**
Complement pathway	31	3.9	<0.001	19.7	**<0.001**
Inflammatory response	27	3.4	<0.001	3.1	**<0.001**
Angiogenesis	23	2.9	<0.001	3.6	**<0.001**
Lipid transport	21	2.6	<0.001	2.7	**<0.001**
Acute phase	17	2.1	<0.001	18	**<0.001**
Complement alternate pathway	13	1.6	<0.001	22.3	**<0.001**
Chemotaxis	12	1.5	0.006	2.6	**0.036**
Glycolysis	10	1.3	<0.001	6	**<0.001**
Cholesterol metabolism	10	1.3	0.003	3.3	**0.020**
Sterol metabolism	10	1.3	0.007	2.9	**0.039**
Steroid metabolism	10	1.3	0.046	2.1	0.230
Stress response	10	1.3	0.067	2	0.310
Cytolysis	8	1	<0.001	9.9	**<0.001**
Notch signaling pathway	8	1	0.009	3.4	0.050
Fibrinolysis	7	0.9	<0.001	19.5	**<0.001**
Oxygen transport	6	0.8	<0.001	9.5	**0.002**
Keratinization	6	0.8	0.029	3.4	0.150
Complement activation lectin pathway	4	0.5	<0.001	22.3	**0.002**

Data were processed by DAVID (the database for annotation, visualization and integrated discovery) tool and arranged in descending order by count of the protein groups related to the specific annotation terms. Only annotation terms with the P values of EASE score lower than 0.05 and minimum of two annotations were included in the table. Corrected P-values from Benjamini-Hochberg correction, with values lower than 0.05, were highlighted in bold. Out of 812 protein groups, 382 were not included in output because of missing annotations.

#### 3.2.1 LIMMA results

**Figure 1:**
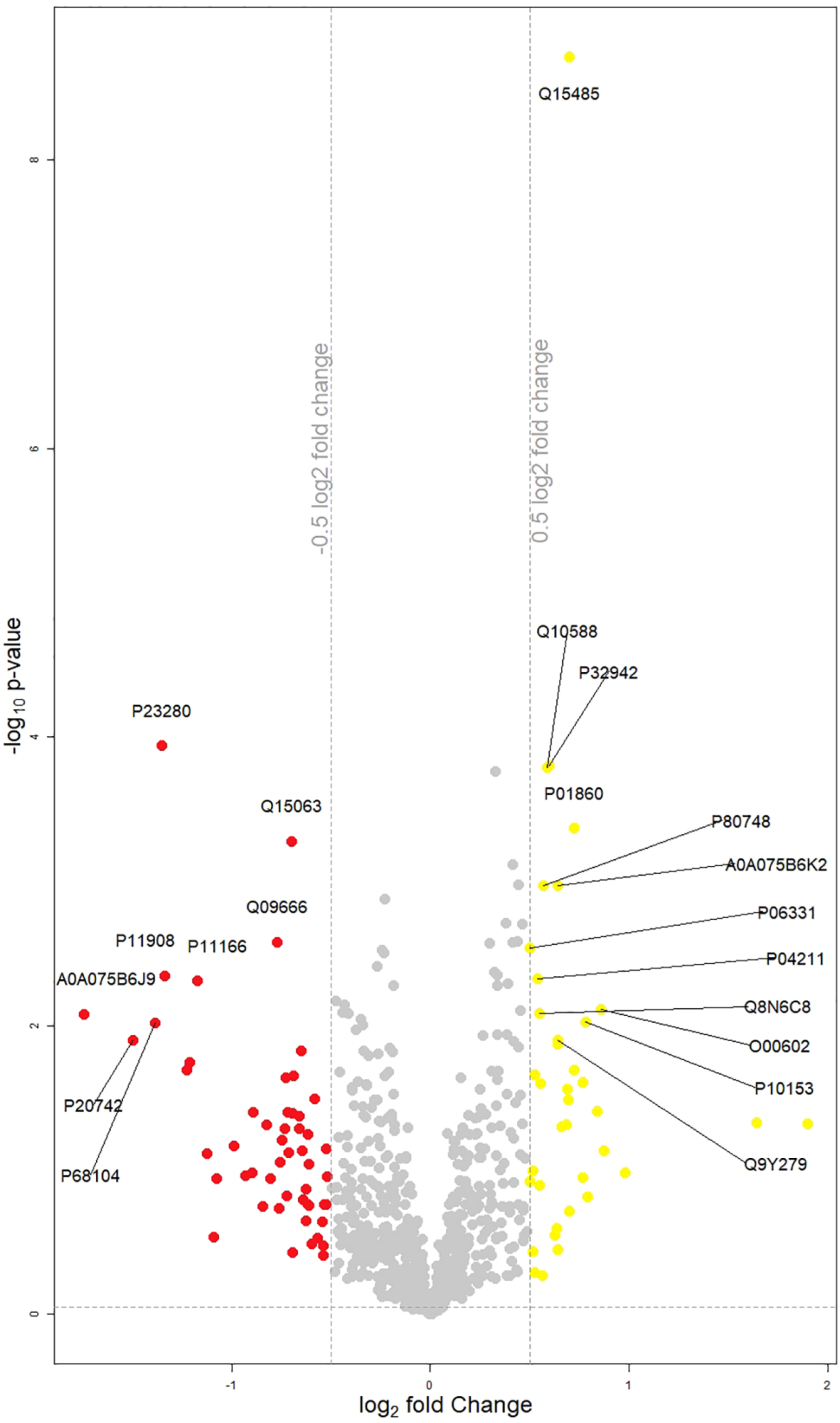
Volcano plot displaying changes in protein groups between week 0 and week 6. Protein groups that are significantly different (p<0.05) and have a log2(fold change) (Week 0/Week 6) of less than -0.5 are highlighted in red, and those with a log2(fold change) greater than 0.5 are highlighted in yellow. Each point represents an individual protein group. Points marked with the corresponding coding gene symbols represent the most significantly up- or down-regulated differentially expressed protein groups.

The LIMMA analysis, performed using both paired and unpaired designs for week 0 and week 6 data, captured several statistically significant findings between selected proteins and clinical variables of interest (MADRS A - D clusters, psychosis, MADRS absolute decrease); models were controlled for sex, age, and BMI. **Figure 1** presents a volcano plot illustrating the dysregulated proteins identified between week 0 and week 6.

Unpaired LIMMA at week 0 found a significant negative association between MADRS cluster C (deficits in cognitive control), alpha-1-antitrypsin (adjusted P value 0.041) and alpha-1-antitrypsin-related protein (adjusted P value 0.041). The same MADRS cluster was in negative association with antileukoproteinase (adjusted P value 0.040) in week 6. In the same week, occurrence of psychosis was associated positively with Macrophage colony-stimulating factor 1 (adjusted P value 0.040).

Paired LIMMA analysis yielded following results: MADRS absolute decrease was negatively associated with complement factor H-related protein 1 (adjusted P value 0.001) and complement factor H-related protein 5 (adjusted P value 0.001) and positively with leukocyte immunoglobulin-like receptor subfamily A member 1 and leukocyte immunoglobulin-like receptor subfamily A member 3 (adjusted. P value 0.021). Deficits in cognitive control (MADRS cluster C) were in positive association with bone morphogenetic protein 1 (adjusted P value 0.004).

#### 3.2.2 WGCNA results

Within the 812 PGs discerned in samples from week 0 and week 6, the WGCNA identified five consensual modules, representing clusters of highly interconnected proteins. ‘Blue’ module included 185 PGs. ‘Brown’ module encompasses 153 PGs. ‘Turquoise’ module consisted of 236 PGs. ‘Yellow’ module involved 134 PGs. ‘Grey’ module comprised ungrouped 104 PGs. Functional annotations of biological processes linked to each module are listed in **Table 5**.

**Table 5:** Biological processes associated with five protein modules compared to all protein groups.

Protein Module	Biological Process	Protein Groups Count	Protein Groups Count (%)	EASE (P-Value)	Fold Enrichment	Benjamini (P-Value)
Blue	Immunity	78	45.6	<0.001	2	**<0.001**
Blue	Adaptive immunity	63	36.8	<0.001	3.1	**<0.001**
Brown	Cell adhesion	30	19.2	<0.001	1.8	**0.025**
Brown	Differentiation	13	8.3	0.004	2.3	0.071
Brown	Transcription regulation	10	6.4	0.041	2	0.390
Brown	Transcription	10	6.4	0.041	2	0.390
Brown	Acute phase	9	5.8	0.012	2.5	0.170
Brown	Notch signaling pathway	7	4.5	0.001	4.2	**0.039**
Turquoise	Host-virus interaction	25	10	0.011	1.6	0.310
Turquoise	Glycolysis	10	4	<0.001	3.9	**0.002**
Turquoise	Stress response	7	2.8	0.020	2.8	0.410
Turquoise	Oxygen transport	6	2.4	0.005	3.9	0.190
Turquoise	Protein transport	6	2.4	0.049	2.6	0.710
Yellow	Transport	18	13.70	0.024	1.7	0.150
Yellow	Blood coagulation	17	13.0	<0.001	3	**<0.001**
Yellow	Hemostasis	17	13.0	<0.001	3	**<0.001**
Yellow	Lipid transport	14	10.7	<0.001	3.9	**<0.001**
Yellow	Lipid metabolism	13	9.9	0.004	2.3	**0.030**
Yellow	Cholesterol metabolism	7	5.3	0.002	4.1	**0.020**
Yellow	Steroid metabolism	7	5.3	0.002	4.1	**0.020**
Yellow	Sterol metabolism	7	5.3	0.002	4.1	**0.020**
Grey	Inflammatory response	9	8.3	0.029	2.3	1.00

Annotations were processed using the DAVID tool (the database for annotation, visualization and integrated discovery). Overrepresentation test and fold enrichment calculations were performed separately for each module with background of all protein groups from our dataset. The functional annotations for each module were sorted in descending order by protein groups count associated with the annotation term. Only annotation terms linked to at least two protein groups and with the P values of EASE score lower than 0.05 were included in the table. Corrected P-values using Benjamini-Hochberg correction, with values lower than 0.05, were highlighted in bold.

##### 3.2.2.1 WGCNA findings at week 0

**Figure 2:**
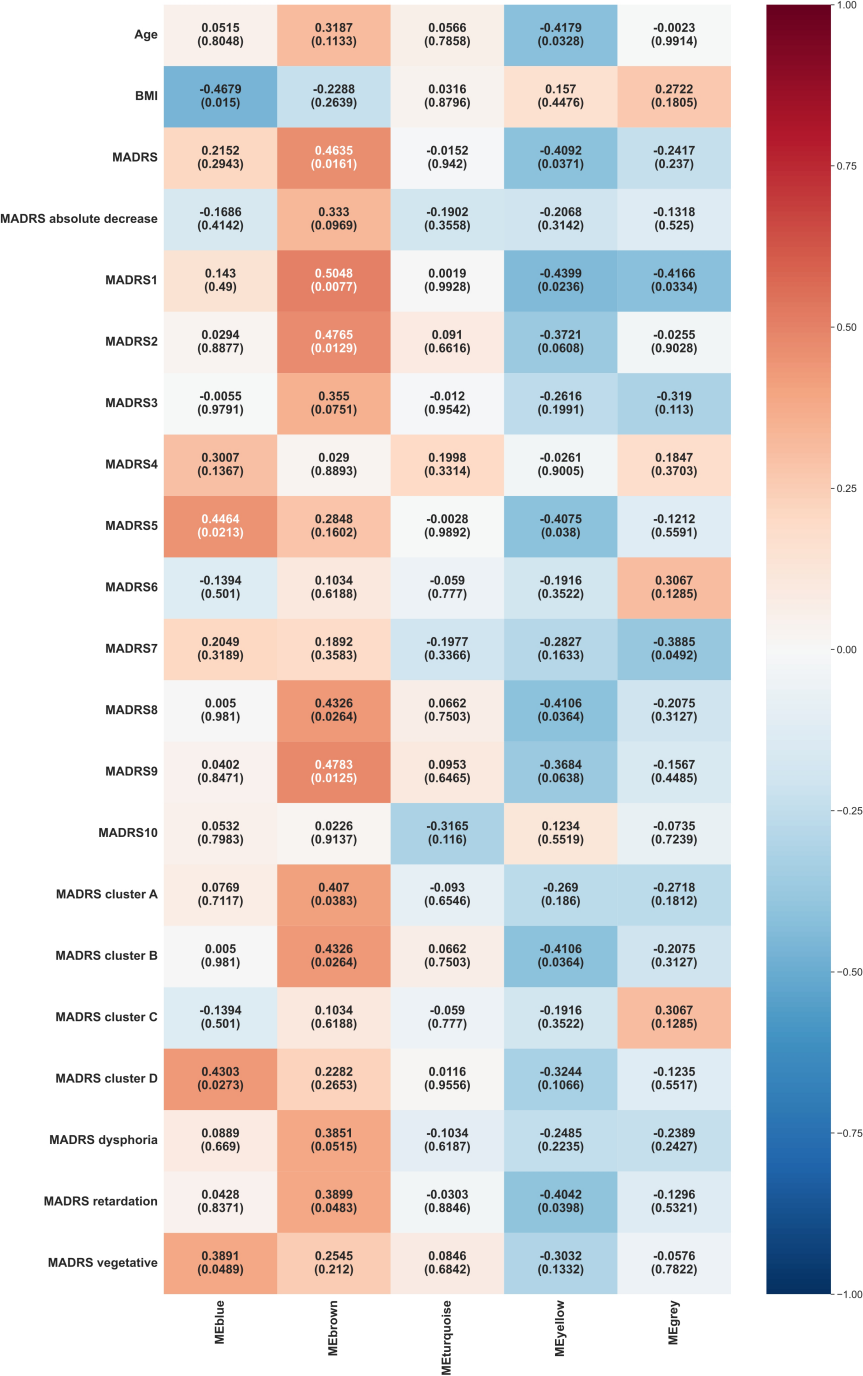
Heat map representation of module-trait relationships at week 0. Each column represents a module eigenvectors, and each row represents demographic/clinical traits. Each cell contains Pearson’s correlation and p-values. The color is coded by correlation with the legend on the right. BMI: body mass index, MADRS: total Montgomery–Åsberg Depression Rating Scale (MADRS) score, MADRS1-MADRS10: MADRS item 1-10 score, MADRS cluster A (Exaggerated reactivity to negative information): MADRS1+MADRS3+MADRS9+MADRS10 score, MADRS cluster B (Altered reward processing): MADRS8 score, MADRS cluster C (Deficits in cognitive control): MADRS6 score, MADRS cluster D (Somatic syndrome): MADRS4+MADRS5+MADRS7 score, MADRS dysphoria: MADRS1+MADRS9+MADRS10 score, MADRS retardation: MADRS2+MADRS6+MADRS7+MADRS8 score, MADRS vegetative: MADRS3+MADRS4+MADRS5 score.

The main WGCNA results from week 0 are displayed in **Figure 2**.

Blue module exhibited significant positive correlations with several MADRS variables, including reduced appetite (MADRS 5, p = 0.0213), somatic syndrome (MADRS cluster D, p = 0.0273), and vegetative factor (MADRS vegetative, p = 0.0489). It also showed a significant negative correlation with BMI (p = 0.015).

Brown module demonstrated significant positive correlations with various MADRS scores, including reported sadness (MADRS 1, p = 0.0077), apparent sadness (MADRS 2, p = 0.0129), inability to feel (MADRS 8, p = 0.0264), pessimistic thoughts (MADRS 9, p = 0.0125), overall MADRS (p = 0.0161), exaggerated reactivity to negative information (MADRS cluster A, p = 0.0383), altered reward processing (MADRS cluster B, p = 0.0264), retardation factor (MADRS retardation, p = 0.0483).

Yellow module showed significant negative correlations with age (p = 0.0328) and various MADRS components, including reported sadness (MADRS 1, p = 0.0236), reduced appetite (MADRS 5, p = 0.038), inability to feel (MADRS 8, p = 0.0364), overall MADRS (p = 0.0371), altered reward processing (MADRS cluster B, p = 0.0364), retardation factor (MADRS retardation, p = 0.0398).

Grey module correlated negatively with reported sadness (MADRS 1, p = 0.0334) and lassitude (MADRS 7, p = 0.0492).

##### 3.2.2.2 WGCNA findings at week 6

**Figure 3:**
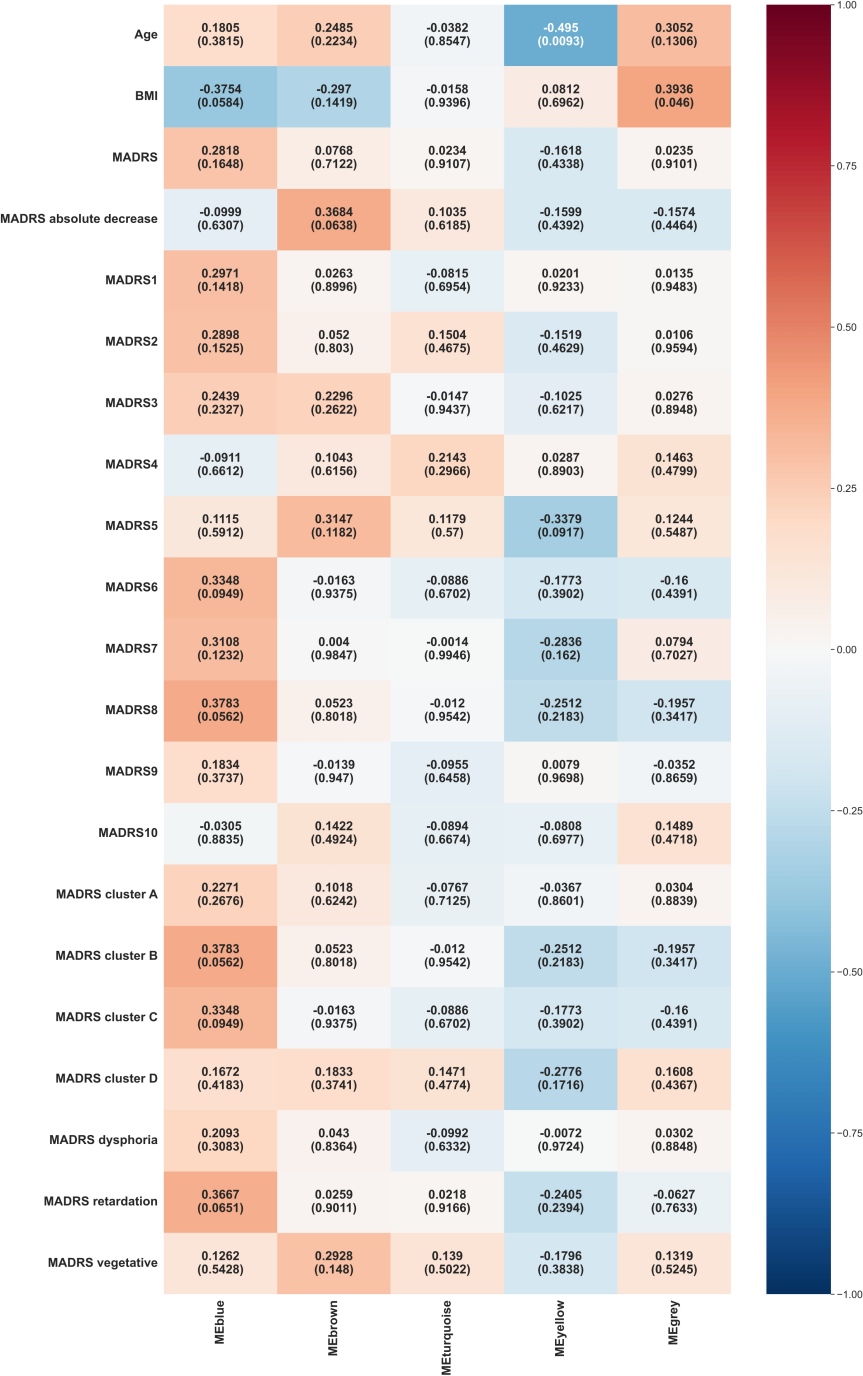
Heat map representation of module-trait relationships at week 6. Each column represents a module eigenvectors, and each row represents demographic/clinical traits. Each cell contains Pearson’s correlation and p-values. The color is coded by correlation with the legend on the right. BMI: body mass index, MADRS: total Montgomery–Åsberg Depression Rating Scale (MADRS) score, MADRS1-MADRS10: MADRS item 1-10 score, MADRS cluster A (Exaggerated reactivity to negative information): MADRS1+MADRS3+MADRS9+MADRS10 score, MADRS cluster B (Altered reward processing): MADRS8 score, MADRS cluster C (Deficits in cognitive control): MADRS6 score, MADRS cluster D (Somatic syndrome): MADRS4+MADRS5+MADRS7 score, MADRS dysphoria: MADRS1+MADRS9+MADRS10 score, MADRS retardation: MADRS2+MADRS6+MADRS7+MADRS8 score, MADRS vegetative: MADRS3+MADRS4+MADRS5 score.

The main WGCNA results from week 6 are presented in **Figure 3**.

Blue module exhibited borderline significant correlations with inability to feel (MADRS 8, p = 0.0562), altered reward processing (MADRS cluster B, p = 0.0562), and BMI (p = 0.0584).

Yellow module continued to show a significant negative correlation with age (p = 0.0093).

Grey module displayed a significant positive correlation with BMI (p = 0.046).

##### 3.2.2.3 WGCNA consensus between week 0 and week 6

**Figure 4:**
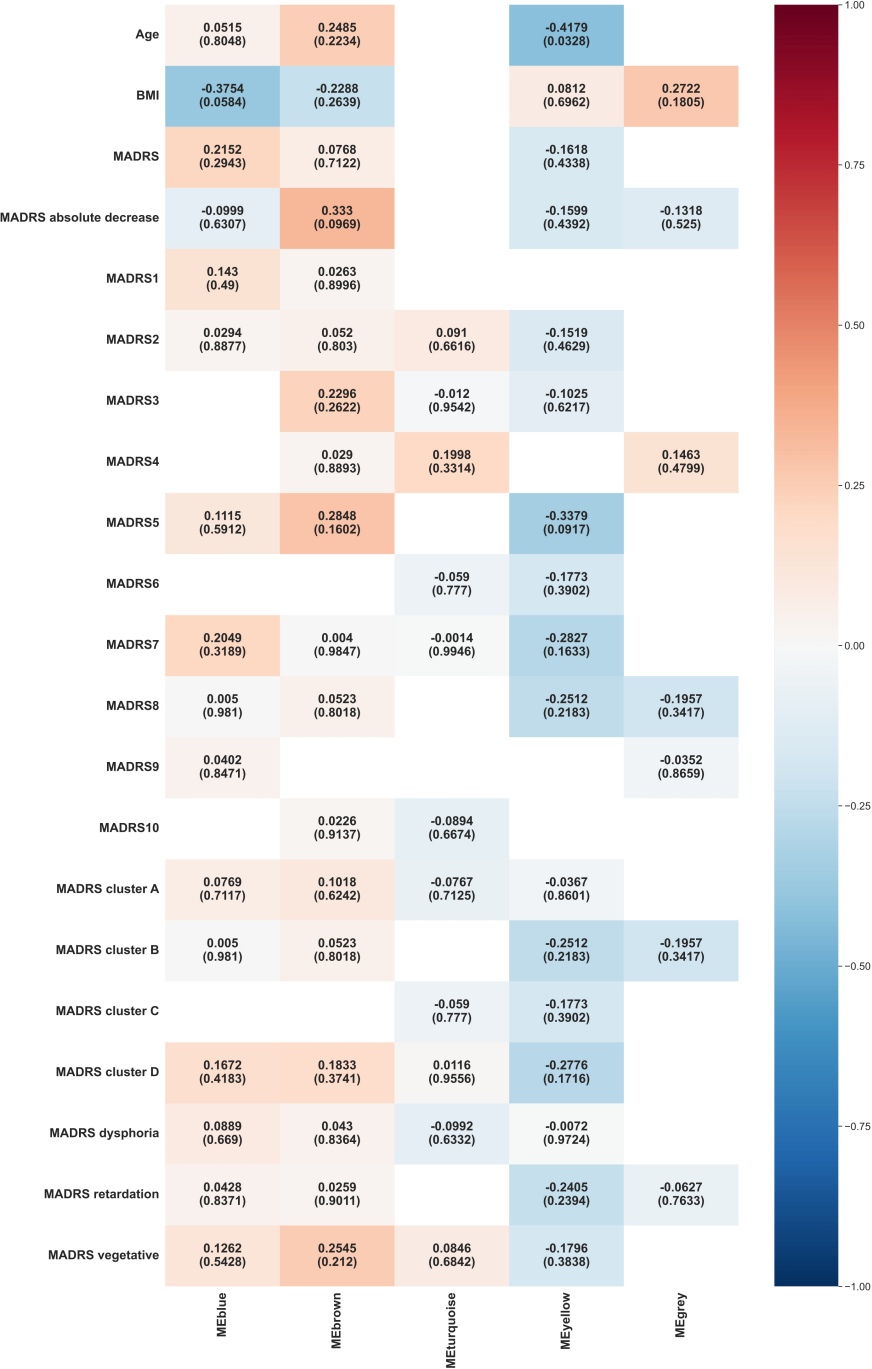
Heat map representation of module-trait relationships across week 0 and week 6. Each column represents a module eigenvectors, and each row represents demographic/clinical traits. Each cell contains Pearson’s correlation and p-values. The color is coded by correlation with the legend on the right. BMI: body mass index, MADRS: total Montgomery–Åsberg Depression Rating Scale (MADRS) score, MADRS1-MADRS10: MADRS item 1-10 score, MADRS cluster A (Exaggerated reactivity to negative information): MADRS1+MADRS3+MADRS9+MADRS10 score, MADRS cluster B (Altered reward processing): MADRS8 score, MADRS cluster C (Deficits in cognitive control): MADRS6 score, MADRS cluster D (Somatic syndrome): MADRS4+MADRS5+MADRS7 score, MADRS dysphoria: MADRS1+MADRS9+MADRS10 score, MADRS retardation: MADRS2+MADRS6+MADRS7+MADRS8 score, MADRS vegetative: MADRS3+MADRS4+MADRS5 score.

The outcomes of the WGCNA consensus analysis are illustrated in **Figure 4**. The modules generally did not show significant consensus between weeks 0 and 6. Blue modules showed a consensus on their negative association with BMI, with a borderline statistical significance (p = 0.0584). Yellow modules demonstrated a consensus on a negative correlation with the age (p = 0.0328).

#### 3.2.3 GLMs analysis

The only clinical variable showing significance at a 0.05 level was achieving remission. The remitters tended to have lower values of blue module eigenvector at week 0 (p = 0.044, beta = -6.262).

#### 3.2.4 RFs analysis

The classifiers demonstrate high efficacy in classification tasks. The impact of the week was negligible for all variables. The sex variable and the first episode were notably influenced by the brown module. The treatment response was affected by both the blue and brown modules. Achieving remission was greatly influenced by the blue module. The occurrence of psychosis was substantially impacted by the yellow module.

## 4 Discussion

This research aimed to elucidate the relationship between the plasma proteome and MDD symptomatology during moderate to severe acute depressive episode, assessing its impact on symptoms and treatment response. Key findings indicate differential associations between specific plasma protein clusters and MDD’s somatic and cognitive-affective symptoms, suggesting varied roles of these clusters in clinical MDD characteristics including first episode manifestation, psychotic depression occurrence, treatment response, and remission achievement.

The biological processes discerned through the analysis of plasma proteins in our patients were primarily engaged in immunity, cell adhesion, host-virus interactions, and hemostasis. A notable overexpression was observed particularly in proteins related to the complement pathway, fibrinolysis, acute phase, hemostasis, cytolysis, oxygen transport, and glycolysis. Such findings align with previous studies of MDD indicating alterations in protein expression profiles, particularly related to immune, inflammatory, and coagulation systems, and further linked to energy metabolism and oxidative stress (11).

A novel aspect of this research involved subclassification of highly co-expressed proteins into five modules, varying in their involvement in critical biological processes. They include:

- Adaptive immunity module (blue module), with the highest proportion of proteins integral to immunity (46%), and particular emphasis on adaptive immunity at 37%.
- Cell adhesion module (brown module) with proteins fundamental to cell adhesion (19.2%), and with overexpression of proteins associated with the Notch signaling pathway.
- Coagulation and lipid transport module (yellow module) distinguished by an unique linkage to proteins engaged in blood coagulation (13%) and lipid transport (10.7%).
- Glycolysis module (turquoise module) with overrepresentation of proteins involved in glycolysis.
- Ungrouped proteins (grey module).

To the best of our knowledge, this is the first investigation to focus on the plasma proteome in MDD symptoms in patients with acute episode achieving these specific outcomes. The only prior proteomic research on MDD symptoms examined plasma proteins via immunoassay in primarily remitted outpatients. Out of six proteomic clusters identified in that study, only one was linked to active depression, specifically to depression with atypical features (37).

### 4.1 Role of adaptive immunity in somatic syndrome, treatment response, and achieving remission

The role of adaptive immunity in somatic syndrome was unveiled by the adaptive immunity module (blue module) that exhibited a positive correlation with both the theoretical construct of a somatic syndrome cluster and a parallel empirical vegetative factor cluster observed during episodes of acute depression. Additionally, this correlation extends to encompass the symptom of diminished appetite, a commonality within both clusters. Such findings lend robust support to the hypothesis that symptomatic manifestations of somatic depression are inextricably linked with inflammatory processes (17, 38, 39). The observed association between the somatic syndrome and specifically adaptive immunity represents a novel finding, although several studies have already indirectly suggested this in melancholic depression, characterized by the prevalence of a somatic syndrome (40–42). In contrast to the somatic symptoms of MDD, the adaptive immunity module revealed an inverse correlation with BMI, a parameter typically found to have a positive association with inflammatory states (43). This observation might substantiate the premise that, notwithstanding the shared pro-inflammatory nature of both obesity and melancholic depression, the nuances of the inflammatory response exhibit marked divergence in these states. This dichotomy in the inflammatory pathways associated with obesity and melancholic depression was previously demonstrated by Martino et al. (2012) in their review of the Th1/Th2 balance, challenging earlier assumptions that in MDD, inflammation is causally linked to adiposity (43, 44).

Alongside the cell adhesion module, the adaptive immunity module significantly contributed to the variability observed in treatment response, with remitters displaying the lowest values of the blue module eigenvector in week 0. This suggests that remitters may exhibit a less active adaptive immune system during acute depression, leading to a reduced propensity for neuroinflammation and neurodegeneration (3, 45). According to another proteomics study in MDD, the difference in treatment response has been primarily attributed to variations in proteins involved in protein metabolism and the immune response. However, in contrast to our results, the proteins linked to the immune response consisted predominantly of those related to the innate immune system (46).

### 4.2 The relationship between cell adhesion, cognitive-affective symptoms, early depression, and treatment response

The cell adhesion module (brown module) exhibited a correlation with exaggerated reactivity to negative stimuli and corresponding items on the MADRS (apparent sadness, reported sadness, and pessimistic thoughts), along with altered reward processing. Furthermore, this module was linked to the retardation factor empirical cluster of MADRS, which encompasses symptoms of avolition such as lassitude and an inability to feel (47).

Whilst the adaptive immunity module was important for achieving remission, the cell adhesion module bore a conspicuous association with the manifestation of the initial episode, according to RFs. Alongside the adaptive immunity module, cell adhesion module had the most substantial impact on treatment response.

Considering cell adhesion’s essential function in cellular communication and regulatory processes, vital for tissue development and maintenance, including that of neuronal tissues, its implicated role across the disorder’s spectrum—from early manifestations to symptom development and response to treatment—appears intuitive (48, 49). However, the role of cell adhesion in treatment response has been demonstrated in only one previous work that revealed the influence of polymorphisms in neuronal cell adhesion genes on response to antidepressants, and studies on the specific role of cell adhesion molecules at the onset of depression, or in the manifestation of specific symptoms of depression, are completely absent (50, 51).

### 4.3 Distinct role of hemostasis and lipid transport in psychotic depression versus other depression symptomatology

In a striking contrast to the positive correlations between the blue and brown modules with the manifestation of depressive symptoms, the coagulation and lipid transport module (yellow module) exhibited an inverse association with such symptoms. This module displayed a non-specific negative correlation with a spectrum of depressive symptoms, encompassing reported feelings of sadness, diminished appetite, and anhedonia. Furthermore, the yellow module was associated with the retardation factor, an empirically derived cluster within the MADRS, encapsulating the MADRS items of lassitude and anhedonia.

The coagulation and lipid transport module emerged as a determinant in the manifestation of psychotic symptoms. Such a link between psychotic depression and proteins involved in hemostasis and lipid transport could be evidenced through a series of notable alterations observed in psychotic depression. These abnormalities, with a potential impact notably on, but not limited to, the coagulation system, include a hyperactivity of the hypothalamo-pituitary axis, an increased uptake of serotonin by platelets, an elevated concentration of serotonin within the platelets, and a higher platelet-lymphocyte ratio (52–54). Additionally, the absence of clear evidence regarding immunity’s specific role in psychotic depression aligns with previous studies. This stands in sharp contrast to the role of immunity in severe depression, treatment resistant depression, and types such as melancholic or atypical depression (17, 52, 53).

### 4.4 Other findings

A LIMMA-based analysis revealed several statistically significant results. Deficits in cognitive control, as indicated by the MADRS item concentration difficulties, exhibited negative correlation with both alpha-1-antitrypsin and alpha-1-antitrypsin-related protein in acute depression, and similarly with antileukoproteinase in the initial stages of remission. Enhancement in cognitive control, observed during the treatment, was positively correlated with the upsurge in bone morphogenetic protein 1. This suggests a nuanced interplay where deficits in cognitive control are paralleled by reduced levels of key enzymes that mitigate tissue damage in inflammatory contexts. Conversely, an improvement in these cognitive aspects aligns with an increase in a biomolecule integral to tissue regeneration and repair processes (55–57).

Macrophage colony-stimulating factor 1 (M-CSF) reached higher levels in patients with psychotic depression during early remission, which may be indicative of a sustained macrophage activation and a pronounced innate immune response within this group (58). Despite the paucity of immunological markers during episodes of depression with psychotic features, as discussed earlier, elevated levels of IL-6 during childhood and adolescence have been associated with an increased risk of developing depression and psychosis in early adulthood (59–61). Such findings, in conjunction with our results, suggest that immune activation could be more of a trait marker rather than a state marker in psychotic depression.

A reduction in the MADRS scores exhibited an inverse correlation with complement components (complement factor H-related protein 1 and complement factor H-related protein 5) and a positive association with immunoglobulin receptors (leukocyte immunoglobulin-like receptor subfamily A member 1, leukocyte immunoglobulin-like receptor subfamily member 3) during the early remission. This may indicate a shift in immune response from innate to adaptive immunity, significant for the decrease in overall depressive symptomatology (62–65). Together with the finding of lower expression of proteins included in the adaptive immunity module in remitters during acute depression, this finding may suggest the importance of a time-delayed activation of adaptive immunity in patients responding well to treatment, maybe due to the enhancement of neuroprotective, or even antidepressant effects mediated by adaptive immunity (3, 45).

### 4.5 Possible intersection of biological processes underlying depression

Several distinct biological processes have been identified as possibly underlying the manifestation of various MDD symptoms and treatment responses, and there are biological mechanisms that may act as common links between them. These include inflammation, oxidative and nitrosative stress (O&NS), and mitochondrial dysfunction (5, 7).

The inflammatory response, often activated as a result of infection, injury, or stress, leads to the release of pro-inflammatory cytokines, such as interleukin-6, tumor necrosis factor-alpha, and interleukin-1 beta. These cytokines modulate the immune response by enhancing immune cell activation and promoting their migration to affected tissues. While this is crucial for defense against pathogens, it can also lead to autoimmune reactions or prolonged inflammatory changes, as may be observed in a significant proportion of MDD patients (3, 5, 66). Pro-inflammatory cytokines regulate the expression of adhesion molecules, which are key for the adhesion of immune cells to the endothelium and their migration to sites of inflammation (67). Additionally, these molecules can influence coagulation by increasing the production of pro-coagulant factors like fibrinogen and reducing the activity of anticoagulant mechanisms (68). Elevated levels of pro-inflammatory cytokines may also disrupt lipid transport (69).

Inflammatory changes are associated with O&NS, the role of which has been indicated by numerous studies on MDD; additionally total antioxidant capacity is decreased in MDD (5, 70). Oxidative stress, often triggered during immune responses, plays a crucial role in modulating these responses, affecting both innate and adaptive immunity. In adaptive immunity, reactive oxygen species can modulate the activation and function of T and B lymphocytes, impacting their ability to respond to specific antigens (71). Oxidative damage to membranes, including the increased expression of neoantigens such as malondialdehyde and oxidized low-density lipoprotein, may lead to the development of chronic inflammatory states or autoimmune reactions (5, 66, 70). Furthermore, O&NS disrupts adhesion molecules, which may weaken cellular contacts, compromise tissue integrity and potentially contribute to pathological states, including inflammatory responses (67). Oxidation of lipids may render them less efficient in transport, which could negatively impact overall lipid metabolism, membranes integrity and cellular function (72). In the context of blood coagulation, oxidative stress can modify lipids and proteins involved in the coagulation process, leading to dysregulated clotting mechanisms (73).

Oxidative stress is strongly linked to mitochondrial disruption, considered one of the potential initiators of the cascade of molecular events leading to MDD. Suboptimal mitochondrial functioning is associated with various processes, including alterations in mitochondrial biochemical cascades, the electron transport chain, and membrane fluidity. Mitochondrial damage, through the activation of apoptotic pathways, may contribute to immune system activation, further exacerbating inflammation and O&NS-related processes (5, 7).

### 4.6 Strengths and limitations

The strengths of this research lie in its execution as a longitudinal study, conducted on a precisely clinically categorized group of markedly ill patients with at least moderate unipolar depression, including individuals with psychotic depression, and availability of biological samples. Evaluations were conducted in a standardized hospital setting of an acute psychiatric ward, ensuring consistent assessment conditions. Diagnoses were established by a team of experienced psychiatrists and were rigorously validated through repeated examinations, including paraclinical methods. Samples from patients in both the acute phase of depression and early remission were incorporated, examining a broad spectrum of plasma proteins, and evaluating numerous clinical characteristics of depression. symptoms. The study utilized innovative statistical methods for data analysis, enhancing the robustness of its findings.

This study’s limitations include a small sample size. The study focused on analyzing proteins in peripheral blood, rather than as central nervous system markers, leaving their cerebral impact partially undefined (74). Protein expression, determined via LC-MS/MS, was assessed on a semi-quantitative basis. In some proteins, annotations were missing. Depressive symptoms were evaluated using tailored MADRS clusters, and while this approach was grounded in existing literature on depression’s core features, it may lack universal applicability. The use of LIMMA in our analysis carries the risk of random false positives from a modest number of protein groups showing positivity out of a total of 812, yet we mitigated this through adjustments with the Benjamini-Hochberg procedure for multiple hypothesis testing (30). The naturalistic design of the study, involving patients undergoing various treatment modalities, might have introduced variability in the findings. Additionally, potential influences from external factors such as lifestyle, medication use, and socio-demographic backgrounds were not fully controlled, though the uniformity of the patient setting within a single medical institution did provide some level of standardization.

### 4.7 Conclusions

This study on plasma proteomics in MDD identified five protein modules, correlating with specific biological processes and uniquely associated with symptom presentation, the disorder’s trajectory, and treatment response. Adaptive immunity correlated with the manifestation of somatic syndrome, cell adhesion was linked to cognitive-affective symptoms, and blood coagulation and lipid transport were predominantly associated with the manifestation of psychotic symptoms. Additionally, cell adhesion molecules played a role in the first episodes of Major Depressive Disorder, both cell adhesion and adaptive immunity were involved in the treatment response, and adaptive immunity was linked to achieving remission.

Such insights suggest the presence of different depression endophenotypes and provide a foundation for future research into targeted therapies and diagnostic markers, potentially leading to more personalized treatment strategies for MDD. Further investigations should encompass larger sample sizes and diverse populations to validate these results.

## Data Availability

The datasets presented in this study can be found in online repositories. The names of the repository/repositories and accession number(s) can be found below: https://figshare.com/, 10.6084/m9.figshare.25719165.
